# Fundamentals and Applications of Analytical Chemistry in Natural Products

**DOI:** 10.1155/2012/156201

**Published:** 2012-07-22

**Authors:** Norberto P. Lopes, Ernani Pinto, Michael Niehues, Paul J. Gates, Ricardo Vessecchi

**Affiliations:** ^1^Núcleo de Pesquisa em Produtos Naturais e Sintético, Faculdade de Ciências Farmacêuticas de Ribeirão Preto, Universidade de São Paulo, 14040-901 Ribeirão Preto, SP, Brazil; ^2^Departamento de Análises Clínicas e Toxicológicas, Faculdade de Ciências Farmacêuticas, Universidade de São Paulo, 05508-900 São Paulo, SP, Brazil; ^3^School of Chemistry, University of Bristol, Cantock's Close, Bristol BS8 1TS, UK

This special issue is aimed at collecting together recent developments in the field of natural product analysis. This issue covers methodologies and applications as diverse as secondary metabolite identification to the detection of metals in food stuffs. The chemistry of natural products has gained prominence not only due to the on-going search for new bioactive substances but also in related areas such as food chemistry and chemical ecology.

We feel that this special issue represents some of the wide ranging research currently performed in this diverse area and this is exemplified by the affiliations of the authors of the papers included which is made up of chemists, biochemists, biologists, physicists, toxicologists, physiologists, pharmaceutists, and geochemists. What all of these researchers have in common, despite their wide ranging backgrounds, is the need to make use of the powerful techniques of analytical chemistry to analyze natural compounds in complex matrices. Thus, this special issue is dedicated to all the readers that would like to apply analytical chemistry for natural products analysis.

This issue presents ten papers, which describe either a specific analytical method for specific types of natural products, the use of natural compounds to aid analytical developments, or modern techniques for the analysis of metals in food stuffs and food quality control (a growing area of interest). 

The article by L. de S. Ferreira et al. describes the use of hyphenated techniques to determine fatty acids in a species of seaweed from the Fernando de Noronha archipelago. In addition, a second paper has used SRTXRF analysis as a technique to determine several inorganic species within the same archipelago. Both papers contribute to an increased understanding of adsorption and accumulation of such natural elements by algae in that specific area.

V. S. A. Devi and V. K. Reddy's paper presents a methodology for spectrophotometric determination of iron(II) and cobalt(II) using 2-hydroxi-1-naphthaldehyde-p-hydroxybenzoichydrazone which can then be applied for the analyses of biological and water samples that contain these metals. 

The paper by G. C. Lopes et al. reports the preliminary estimation of the stability of the dried extract from the bark of *Guazuma ulmifolia Lam*. (“Mutamba”). Thermogravimetry analysis along with HPLC were used in the study. The results can be used as a chemical marker in the quality control of dried extracts of *G. ulmifolia*. 

The study by M. F. Zampa et al. is the first report of the antimicrobial peptide from the skin of the *Phyllomedusa hypochondrialis* frog and its incorporation into nanostructured layered films in conjunction with nickel-tetrasulfonated phthalocyanines. The film was used as a biosensor to detect dopamine, a neurotransmitter associated with diseases such as Alzheimer's and Parkinson's. 

S. B. A. Barros et al. demonstrate the exploitation of the polyelectrolytic behavior of natural cashew gum (*Anacardium occidentale L.*), found in northeast Brazil, as a component of a nanocomposite electrode. The performance of the electrodes was evaluated by the determination of dopamine. 

The paper by H. Tanaka et al. presents matrix-assisted laser desorption/ionization (MALDI) mass spectrometry for the confirmation of the hapten number in synthesized antigens. Two unique applications using MAb, Eastern blotting, and knockout extract have been introduced in this paper. The Eastern blotting method has great potential applications for the wide range of natural products. 

The article of S. R. de Moraes et al. highlights the use of *Lippia sidoides Cham*. (also known as alecrim pimenta), native to northeastern Brazil and northern Minas Gerais, and their essential oils. The oxygenated monoterpene 1,8-cineole was the major constituent, followed by isoborneol and bornyl acetate. The chemical composition of essential oils described in this paper differs from that described in the literature for *L. sidoides *found in its native environment. 

The paper of M. J. Kato et al. demonstrates the GC-MS monitoring of the major secondary metabolites and fatty acids occurring in the seeds of *Virola surinamensis,* during germination and seedling development. The authors conclude that the germination of *V. surinamensis *seeds and the seedling development are processes in which both fatty acids and secondary metabolites (lignans, isoflavonoids and juruenolides) are equally consumed in the seeds indicating their potential physiological role as energy and carbon sources.

The article of P. J. Gates and N. P. Lopes describes the application of negative ion chip-based nanospray tandem mass spectrometry for the analysis of flavonoid aglycones. The methodology is tested by the analysis of a crude green tea extract, where the expected flavonoids were readily identified.

*Norberto P. Lopes* *Norberto P. Lopes* *Ernani Pinto* *Ernani Pinto* *Michael Niehues* *Michael Niehues* *Paul J. Gates* *Paul J. Gates* *Ricardo Vessecchi* *Ricardo Vessecchi*

